# Placental Pathogens Associated With Adverse Maternal and Neonatal Outcomes

**DOI:** 10.1093/ofid/ofag229

**Published:** 2026-04-22

**Authors:** Alicia Liu, Caitlin Little, Talia Dubowitz, Malinda Schaeffer, Lauren Skvarca, Anne-Marie Rick, Thomas Hooven, Michael Morowitz, Sharon L Hillier, Harold C Wiesenfeld, Christina J Megli

**Affiliations:** Department of Obstetrics, Gynecology and Reproductive Sciences, University of Pittsburgh School of Medicine, Pittsburgh, Pennsylvania, USA; Magee-Womens Hospital of UPMC, Pittsburgh, Pennsylvania, USA; Department of Obstetrics, Gynecology and Reproductive Sciences, University of Pittsburgh School of Medicine, Pittsburgh, Pennsylvania, USA; Department of Obstetrics and Gynceology, Geisinger Medical Center, Danville, Pennsylvania, USA; Magee-Womens Research Institute, Pittsburgh, Pennsylvania, USA; Department of Obstetrics and Gynecology, University of Iowa, Iowa City, Iowa, USA; Department of Pathology, Magee-Womens Hospital of UPMC, Pittsburgh, Pennsylvania, USA; Department of Pediatrics, University of Pittsburgh School of Medicine, Pittsburgh, Pennsylvania, USA; Department of Pediatrics, University of Pittsburgh School of Medicine, Pittsburgh, Pennsylvania, USA; Department of Surgery, University of Pittsburgh School of Medicine, Pittsburgh, Pennsylvania, USA; Department of Obstetrics, Gynecology and Reproductive Sciences, University of Pittsburgh School of Medicine, Pittsburgh, Pennsylvania, USA; Magee-Womens Hospital of UPMC, Pittsburgh, Pennsylvania, USA; Magee-Womens Research Institute, Pittsburgh, Pennsylvania, USA; Department of Obstetrics, Gynecology and Reproductive Sciences, University of Pittsburgh School of Medicine, Pittsburgh, Pennsylvania, USA; Magee-Womens Hospital of UPMC, Pittsburgh, Pennsylvania, USA; Magee-Womens Research Institute, Pittsburgh, Pennsylvania, USA; Department of Obstetrics, Gynecology and Reproductive Sciences, University of Pittsburgh School of Medicine, Pittsburgh, Pennsylvania, USA; Magee-Womens Hospital of UPMC, Pittsburgh, Pennsylvania, USA; Magee-Womens Research Institute, Pittsburgh, Pennsylvania, USA

**Keywords:** maternal sepsis, neonatal sepsis, placenta, maternal-fetal interface, pregnancy

## Abstract

**Background:**

Bacterial infection at the maternal-fetal interface contributes to adverse pregnancy outcomes, including preterm birth, chorioamnionitis, and sepsis. Despite evolving obstetric practices that may influence microbial patterns, recommended clinical algorithms for prophylaxis and treatment rely on outdated data. Furthermore, the pathogenic organisms associated with adverse outcomes remain incompletely characterized.

**Methods:**

We conducted a retrospective study of placental cultures from a high-risk obstetric cohort from a single hospital system between 2014 and 2019 (n = 714). Placental cultures were obtained from deliveries at high risk for infection: (eg, preterm birth or suspected chorioamnionitis). Placental cultures were stratified into pathogenic and nonpathogenic. Associated clinical outcomes, histopathology, and antimicrobial susceptibility patterns were compared.

**Results:**

Pathogenic organisms were identified in 69% of cultures (492 of 714). Group B *Streptococcus* (n = 161; 22.5% of total isolates) and *Escherichia coli* (n = 53; 7.4% of total isolates) were the most common pathogens isolated; we also identified additional pathogens, including *Enterococcus faecalis* (n = 44), *Haemophilus* (n = 37), *Prevotella* (n = 32), and *Bacteroides* species (n = 23). Pathogenic cultures were correlated with significantly higher rates of composite adverse maternal (27.0% vs 17.6%; *P* = .006) and neonatal (78.9% vs 60.6%; *P* < .001) outcomes, as well as histopathologic chorioamnionitis (72.5% vs 60.9%; *P* = .003). Antimicrobial resistance for recommended empiric regimens ranged from 47.1% to 87.8%.

**Conclusions:**

Bacterial pathogens isolated from the placenta in high-risk pregnancies are associated with perinatal morbidity. Group B *Streptococcus* and *E coli* remain common, and *E faecalis*, *Haemophilus* spp, and anaerobes such as *Prevotella* may represent emerging pathogens underrecognized in contemporary obstetric practice. Placental cultures may serve as a valuable surveillance tool for informing empiric antibiotic strategies in pregnancy.

Ascending bacterial infections at the maternal-fetal interface are associated with significant adverse outcomes, including preterm labor, preterm premature rupture of membranes (PPROM), chorioamnionitis, and intrauterine fetal demise (IUFD) [1–4]. Infection remains the second leading cause of death in pregnancy and maternal morbidity from infection despite the availability of antibiotics [5], underscoring key gaps in understanding the pathogenesis and management of obstetric infections.

The microbiology of ascending infections during pregnancy was characterized in the 1980s and 1990s, using amniotic fluid obtained by amniocentesis [6–8]. Amniocentesis is no longer recommended for intrauterine infection, and the frequency of pathogens and associated antimicrobial resistance patterns are not routinely monitored. Current empiric treatment algorithms rely on historical pathogen profiles that may not reflect the organisms encountered in modern obstetric care [9, 10]. Notably, many of these antibiotic regimens were established prior to key changes in obstetric care, including screening and prophylaxis for universal group B *Streptococcus* (GBS; *Streptococcus agalactiae*) and the use of latency antibiotics for PPROM [11–13]. These interventions may have altered the microbial landscape and resistance profiles at the maternal-fetal interface.

The drivers of antibiotic resistance in the obstetric population may differ significantly from those in general medical or surgical cohorts and are largely uncharacterized [14, 15]. Some study findings suggest that the perinatal microbiologic landscape may be shifting with increased resistance in gram-negative pathogens: early-onset neonatal sepsis, which is strongly linked to intrauterine infection and vertical transmission of bacterial pathogens, has shown a decline in GBS as a causative pathogen, with *Escherichia coli* emerging as a more prominent cause of early-onset neonatal sepsis [16–19]. Blood cultures from pregnant patients with bacteremia found a significant enrichment of *E coli* resistant to ampicillin and extended spectrum β-lactams (ESBLs) [20] However, because bacteremia is rare in pregnancy, these data underrepresent the broader burden of intrauterine infection and do not capture organisms of lower prevalence.

Placental cultures, obtained after delivery, offer a practical approach for surveillance of bacterial pathogens and antimicrobial resistance at the maternal-fetal interface [21, 22]. Currently, the clinical utility of placental cultures remains uncertain and these cultures are not routinely incorporated into obstetric management. Findings of some studies suggest that the placental microbiome reflects tissue contamination rather than colonization in healthy pregnancies [26, 27, 29], but the utility of traditional, culture-based, methods in high-risk settings is unknown. Limited data suggest that positive placental cultures in cases of clinical chorioamnionitis are associated with worse neonatal outcomes [21], but their role in guiding treatment or predicting complications remains unclear. While placental cultures are not routine in obstetric practice, our center sends placental cultures in pregnancies at risk for ascending infection, creating an opportunity to evaluate the microbiology of placental microorganisms in clinical scenarios with a high risk of infection (suspected chorioamnionitis/intrauterine infection or preterm birth).

In this retrospective cohort study of 714 patients with positive placental cultures, we sought to define the modern microbial landscape of the maternal-fetal interface and its clinical implications. Our primary objective was to identify pathogens associated with adverse maternal and neonatal outcomes and evaluate the relationship between microbiologic isolates, placental histopathology, and clinical morbidity. As a secondary objective, we examined species-specific patterns of disease severity and antimicrobial resistance—including the potential emergence of underrecognized pathogens of obstetric importance. By integrating microbiologic, clinical, and histopathologic data, this study addresses critical knowledge gaps in the surveillance of intrauterine infection and provides evidence to inform empiric antimicrobial strategies for the mother–infant dyad.

## METHODS

### Study Design and Population

We conducted a retrospective cohort study of all obstetric patients with positive placental cultures processed through the centralized microbiology laboratory of the University of Pittsburgh Medical Center system between 1 January 2014 and 31 December 2019. Per institutional practice, placental cultures are obtained after delivery in clinical contexts concerning for intrauterine infection or inflammation, including suspected chorioamnionitis, preterm delivery, prolonged rupture of membranes (>18 hours), and IUFD. All cultures demonstrating microbial growth were eligible for inclusion. Exclusion criteria included gestational age <14 weeks, confirmed Toxoplasma, Other (Zika), Rubella, CMV or Herpes (TORCH) infections of the placenta, or missing clinical data.

### Patient Consent Statement

This study was approved by the University of Pittsburgh Institutional Review Board (STUDY20060145) with waiver of informed consent due to the retrospective design and analysis of deidentified data.

### Placental Culture Identification and Microbiologic Processing

This study did not involve collection of research specimens. Instead, all placental cultures were obtained as part of routine clinical care, and the study cohort was identified retrospectively from positive placental culture reports generated by the University of Pittsburgh Medical Center central microbiology laboratory. Per institutional protocol, placental cultures are collected by trained nursing staff immediately after delivery in clinical scenarios concerning for intrauterine infection or inflammation. Swab samples from the maternal and fetal placental surfaces are obtained using sterile technique and processed by the microbiology laboratory using standard aerobic and anaerobic culture methods. Organisms are identified using conventional biochemical assays and matrix-assisted laser desorption/ionization–time of flight mass spectrometry when available.

### Clinical Data Abstraction

Maternal and neonatal demographic and clinical data were manually extracted from linked electronic health records by trained research personnel. Data abstraction included patient characteristics, obstetric history, delivery outcomes, and maternal and neonatal morbidity (see Supplementary Table 1). All data were reviewed for accuracy and clinical relevance by maternal-fetal medicine specialists (C. J. M. and M. S.). Placental histopathology results were abstracted from the record (when available) and characterized by a dedicated perinatal pathologist (L. S.) into maternal and neonatal inflammation, using the Amsterdam criteria [30]. This classification occurred independent of access to clinical and microbiologic outcomes. All patient data were deidentified before analysis.

### Definitions Used in This Study

IUFD was defined as fetal death diagnosed before the time of delivery at ≥20 weeks 0 days of gestation. Previable delivery was defined as birth before 23 weeks 0 days of gestation, the threshold for neonatal resuscitation per institutional protocols during this time period. Preterm birth was defined as live birth between 20 weeks 0 days and 36 weeks 6 days of gestation. Maternal morbidity and neonatal morbidity were both measured using respective composite scores. Severe maternal morbidity was defined as the presence of ≥1 of the following: postpartum wound infection with hospital admission, intra-abdominal abscess, endometritis with hospital admission, postpartum reoperation, need for >1 uterotonic agent, intrauterine balloon placement, intensive care unit (ICU) admission, mechanical ventilation or vasopressor support, and/or maternal death.

Composite severe neonatal morbidity was scored for any neonate who met the following criteria: unexpected neonatal ICU admission (eg, term neonate), neonatal sepsis defined as positive blood cultures or treatment with >7 days of antibiotics initiated within 48 hours of birth, necrotizing enterocolitis, or neonatal death. Neonatal ICU admission was defined as admission of a neonate born at ≥23 weeks’ gestation who was resuscitated and survived beyond delivery, excluding those managed with comfort care only. Antepartum antibiotic administration includes antibiotics given during the delivery hospitalization before delivery, including for PPROM, suspected chorioamnionitis, GBS prophylaxis, preoperative antibiotics administration, other diagnosed infection during the hospitalization, and maternal sepsis.

### Classification of Bacterial Pathogens

Bacterial culture results were stratified into pathogenic, nonpathogenic, and indeterminate by expert review (C. J. M., S. H., and H. C. W.). Pathogenic included any culture result with GBS, *E coli, Haemophilus* spp, *Klebsiella* spp, *Enterococcus faecalis*, *Bacteroides* spp, *Bacillus spp*, *L monocytogenes*, *Eikenella* sp, *Citrobacter* sp, *Fusobacterium*, group A *Streptococcus* (*Streptococcus pyogenes*), *Proteus mirabilis, Pseudomonas aeruginosa*, *Enterobacter* spp, *Serratia marcescens*, *Neisseria gonorrhoeae,* or *Clostridium perfringens.* Nonpathogenic bacteria included designation as “normal vaginal flora,” *Lactobacillus* spp, *Pepto streptococcus*, *Peptoniphilus, Mobiluncus, Propionibacterium, Bifidobacterium,* or nonpathogenic *Staphylococcus* and *Streptococcus* species (eg, group D streptococci, nonspeciated α-hemolytic streptococci, viridans streptococci and coagulase-negative staphylococci, and *Staphylococcus epidermidis*). The indeterminate category included *Streptococcus anginosus, Streptococcus gallolyticus, Prevotella* spp, *Candida albicans*, and *Gardnerella vaginalis*. If a placental culture result included both a pathogenic organism and a nonpathogenic organism (eg, *E coli* and *Lactobacillus* spp), the patient was designated as having a pathogenic culture.

### Antimicrobial Susceptibility

Antimicrobial susceptibility of bacterial isolates was abstracted from the microbiology report. Because antimicrobial susceptibility testing is not comprehensive on all isolates, when these were absent presumptive susceptibilities were assigned based on species-level expectations, using the *Sanford Guide to Antimicrobial Therapy* [31]. Key assumptions included the following: *Bacillus* spp are sensitive to clindamycin and vancomycin; *Bacteroides* spp are sensitive to ampicillin-sulbactam and resistant to cefazolin; *Prevotella* spp are sensitive to clindamycin, cefoxitin, and cefotetan and resistant to cefazolin and vancomycin; *Actinomyces* is sensitive to cefoxitin, ampicillin, clindamycin, and ampicillin-sulbactam and resistant to cefotetan and vancomycin; and *E faecalis* is resistant to cephalosporins, gentamicin, and clindamycin.

### Statistical Analysis

Statistical analysis was performed using GraphPad Prism software version 10. All analyses were univariate. Continuous variables were compared using unpaired Student *t* tests, dichotomous variables were using χ^2^ tests, as appropriate. Differences were considered statistically significant at *P* < .05. Descriptive statistics were given as frequencies with percentages or means with standard deviations.

### Secondary Analysis: Identification of Emerging or High-Risk Pathogens

To explore the association between specific pathogens and adverse clinical outcomes, we conducted a secondary analysis comparing maternal and neonatal outcomes stratified by individual pathogenic species. Pathogens were considered of potential emerging or underrecognized clinical importance if they were both (1) associated with significantly increased maternal or neonatal morbidity and (2) less frequently reported in historical cohorts or considered uncommon in obstetric infections. The frequencies of each pathogen were calculated, and outcomes were compared, using χ^2^ or Fisher exact test as appropriate. Organisms meeting the above criteria were flagged for further consideration as potential emerging or underrecognized threats to maternal or neonatal health. These findings were interpreted in the context of both organism frequency and associated clinical outcomes.

## RESULTS

### Cohort Identification and Characteristics

A total of 729 patients with positive placental cultures were identified by microbiology reports; 15 were excluded (delivery at ≤14 weeks’ gestational age, confirmed TORCH infection, or missing clinical data), yielding a final cohort of 714 patients. This cohort reflected the high-risk characteristics of indications for placental cultures including high rates of stillbirth (16.3%), suspected chorioamnionitis (33.6%), with a mean gestational age at delivery of 27.5 weeks. Antepartum antibiotics were administered to 59.6% of patients.

### Pathogen Distribution and Reclassification of Indeterminate Organisms

**Figure 1.**
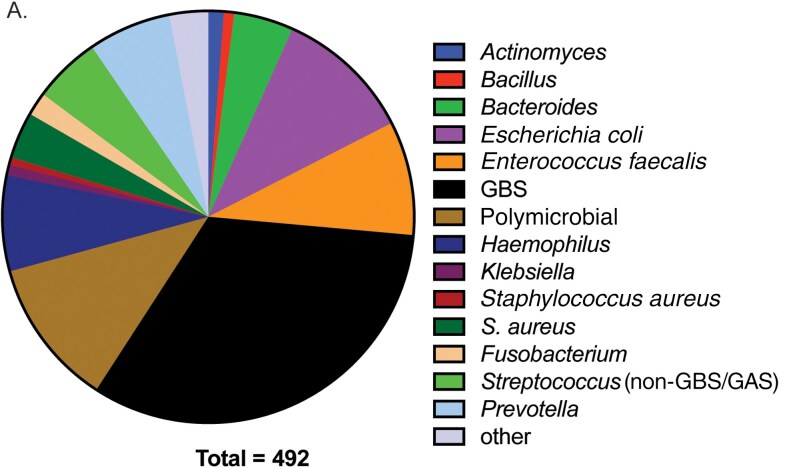
Composition of pathogenic bacterial cultures. Abbreviations: GAS, group A *Streptococcus*; GBS, group B *Streptococcus*.

Pathogenic isolates most frequently included GBS, *E coli*, *E faecalis*, *Haemophilus* spp, *Bacteroides* spp, and *Prevotella* spp (Figure 1). Polymicrobial cultures comprised 11.5% of pathogenic isolates. *Prevotella* spp, *S anginosus*, and *S gallolyticus* (classified as indeterminant) were reclassified as pathogenic based on earlier gestational age at delivery and high rates of histopathologic inflammation. In contrast, *G vaginalis* and *C albicans* were associated with low inflammation and later gestational age at delivery and were categorized as nonpathogenic. After this classification, 492 placental cultures (68.9%) were classified as pathogenic and 222 (31.1%) as nonpathogenic (Figure 1, Supplementary Table 2, and Supplementary Figure 1).

### Maternal and Neonatal Outcomes

Maternal demographics and baseline characteristics were similar between pathogenic and nonpathogenic groups, including maternal age, parity, history of prior preterm birth, and receipt of antibiotics before delivery (Table 1). Indications for culture, delivery mode and gestational age at delivery were different (Table 1). Pathogenic cultures were associated with higher rates of composite maternal morbidity (27.0% vs 17.6%; *P* = .006), primarily driven by increased endometritis or wound complications (8.9% vs 2.4%; *P* = .003) and maternal sepsis (4.9% vs 0.9%, *P* = .009) (Table 2). Markers of additional severe maternal morbidity, including maternal ICU admission and maternal intubation and/or pressor support, were increased with pathogenic placental culture (5.7% vs 2.3% [*P* = .04] and 2.6% vs 0.45% [*P* = .05], respectively) (Table 2). Maternal hemorrhage rates did not differ (Table 2).

**Table 1.** Maternal Demographic and Baseline Characteristics

Characteristic	Mothers, % (No.)^a^					*P* Value
	All (n = 714)			Pathogen, (n = 492)	Nonpathogen (n = 222)	
Age, mean (SD), y	28.0 (6.1)			28.3 (5.9)	27.9 (6.16)	.36
Multiparous	61.4 (438)			61.5 (304)	61.3 (136)	.94
Parity, mean (SD)	0.86 (1.2)			0.92 (1.2)	0.84 (1.2)	.44
History of preterm birth	18.4 (109^e^)			26.5 (81)	21.3 (29)	.24
Indication for placental culture^b^	94.8 (677)			95.1 (470)	94.1 (209)	.58
Antibiotics administered before delivery	59.6 (427)			61.4 (302)	56.3 (125)	.20
Penicillin allergy	10.2 (73)			10.1 (50)	10.4 (23)	.92
Induction of labor	38.9 (244^f^)			39.8 (176)	37.2 (68)	.54
GA, mean, d^c^	194.2	198.1	194.1	198.3	185.3	.008^d^
Maternal bacteremia	2.9 (22)			3.3 (15)	2.7 (6)	.69
Stillbirth (GA >20 wk)	16.3 (117)			15.0 (74)	19.4 (43)	.15
Mid trimester loss (14–20 wk)	22.1 (158)			20.1 (99)	26.6 (59)	.05
Suspected IUI	33.6 (240)			39.2 (193)	21.2 (47)	<.001^d^
Preterm birth at >20 wk	38.0 (271)			38.4 (189)	36.9 (82)	.71
Multiple indications	18.7 (134)			21.3 (105)	13.1 (29)	.009^d^
Term delivery	25.4 (182)			28.3(139)	19.4 (43)	.01^d^
Mode of delivery						<.001^d^
Vaginal	66.6 (476)			66.1 (325)	68.0 (151)	…
Cesarean	21.1 (151)			24.6 (121)	13.5 (30)	…
D&E	12.2 (87)			9.4 (46)	18.5 (41)	…

Abbreviations: D&E, dilation and evacuation; GA, gestational age; IUI, intrauterine infection.

^a^Data represent % (no.) unless otherwise specified.

^b^Indication for placental culture indicates that a clinical adverse outcome documented in the clinical record met institutional criteria to send for placental culture.

^c^GA at completion of pregnancy.

^d^Significant at *P* < .05.

**Table 2.** Maternal Outcomes Associated With Positive Placental Culture

Outcome	Pathogen, % (No.)	Nonpathogen, % (No.)	*P* Value
Composite adverse maternal outcome^a^	27.0 (133)	17.6 (39)	.006^b^
Endometritis/infectious wound complications^c^	8.9 (44)	2.7 (6)	.002^b^
Reoperation or postpartum return to OR^d^	11.2 (55)	6.8 (15)	.07
Retained products	9.8 (48)	6.8 (15)	.19
Hysterectomy	0.81 (4)	0	.2
ICU admission	5.7 (28)	2.3 (3)	.04^b^
Intubation or pressor support	2.64 (13)	0.45 (1)	.05
Maternal death	0.20 (1)	0	.5
Sepsis	4.9 (24)	0.9 (2)	.009^b^
EBL >1000 mL	13.0 (64)	18.9 (23)	.10
>1 Uterotonic	17.9 (88)	14.9 (33)	.32
Balloon placement	1.0 (5)	0	.13

Abbreviations: EBL, estimated blood loss; ICU, intensive care unit; OR, operating room.

^a^Composite adverse maternal outcome defined as postpartum wound infection with hospital admission, intra-abdominal abscess, endometritis with hospital admission, postpartum reoperation, need for >1 uterotonic agent, intrauterine balloon placement, ICU admission, mechanical ventilation or vasopressor support, and/or maternal death.

^b^Significant at *P* < .05.

^c^Pregnancy complicated by a clinical diagnosis of postpartum endometritis or wound complications, defined as wound dehiscence, cellulitis, or wound infection requiring treatment.

^d^Return to the OR for a repeated procedure or unanticipated OR procedure in the setting of a vaginal delivery.

**Figure 2.**
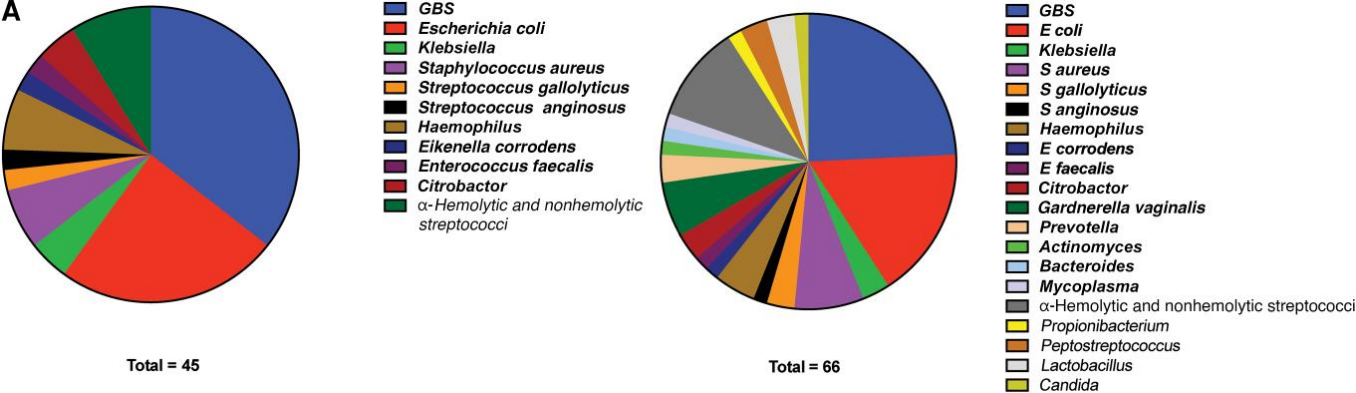
Placental cultures that correspond with positive neonatal blood culture *A,* Concordant (*A*) and discordant (*B*) neonatal and placental cultures. Bolded text indicate organisms categorized as pathogens. Abbreviation: GBS, group B *Streptococcus*.

Composite neonatal morbidity was higher with pathogenic cultures (78.4% vs 60.6%; *P* < .001) (Table 3). Neonatal antibiotic treatment (86.1% vs 43.1%) and clinical neonatal sepsis (56.0% vs 36.9%) were significantly increased in the pathogenic group (*P* < .001). Positive neonatal blood cultures were uncommon overall; however, when present, 83.0% were concordant with the maternal pathogenic placental isolate (Figure 2 and Supplementary Table 3). Neonatal cultures associated with nonpathogenic placental isolates were largely discordant (Supplementary Table 4).

**Table 3.** Fetal or Neonatal Outcomes Associated With Pathogenic Placental Culture

Outcome	Pathogen, % (No.) (n = 260)	Nonpathogen, % (No.) (n = 104)	*P* Value
Composite adverse neonatal outcome^a^	78.9 (205)	60.6 (62)	<.001^b^
NEC^c^	8.4 (20)	6.1 (6)	.48
Neonatal antibiotics^d^	86.1 (216)	43.1 (87)	<.001^b^
Neonatal sepsis at <7 d of life^e^	56.03 (144)	36.9 (38)	<.001^b^
Positive neonatal blood culture <2 h after delivery	19.9 (52)	17.5 (18)	.59
Death	8.1 (21)	5.8 (6)	.46
Unanticipated death^f^	7.4 (19)	4.9 (5)	.39

Abbreviation: NEC, necrotizing enterocolitis

^a^Composite adverse neonatal outcome defined as unexpected neonatal intensive care unit admission (eg, in a term neonate), neonatal sepsis (defined as positive blood cultures or treatment with >7 days of antibiotics initiated within 48 hours of birth), NEC, or neonatal death.

^b^Significant at *P* < .05.

^c^Denominator calculated using infants who remained alive at 48 hours.

^d^Any neonatal antibiotics administered for ≥24 hours.

^e^Neonatal sepsis occurring at <7 days of life, defined as a positive blood culture or treatment of neonatal sepsis with >7 days of antibiotics initiated within 48 hours of birth.

^f^Unanticipated death in a neonate in whom comfort measures were not initiated after birth.

### Placental Histopathology and Inflammation Stratification

**Figure 3.**
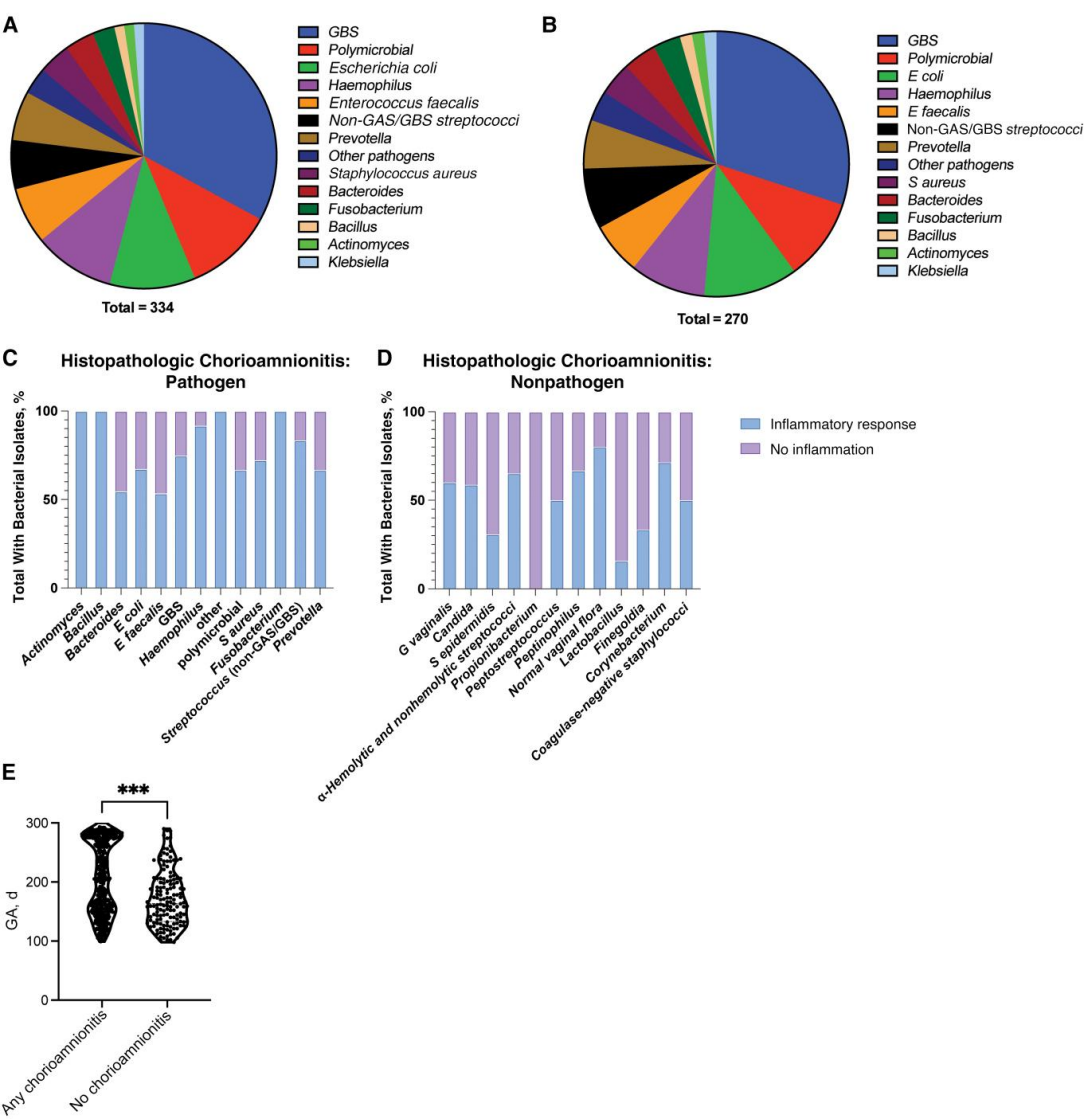
Placental culture results as stratified by histopathology results. *A*. Frequency of all placental culture results with histopathologic evidence of maternal or fetal inflammatory response by Amsterdam criteria. Abbreviations: GAS, group A *Streptococcus*; GBS, group B *Streptococcus*. *B*, Frequency of placental pathogens in placental culture results with histopathologic evidence of fetal inflammatory response. *C, D,* Frequency of histopathologic evidence of chorioamnionitis as stratified by pathogen (*C*) or nonpathogen (*D*) bacterial designation. *E,* Gestational age (GA) at delivery with histopathologic chorioamnionitis versus no chorioamnionitis. ***P < .001.

Histopathology was available for 94% of placentas in the pathogenic group and 85% in the nonpathogenic group. Histologic chorioamnionitis was more common in the pathogenic group (72.3% vs 51.0%; *P* < .001) (Figure 3*A*–3*D*). Among placentas with pathogenic isolates and inflammation, maternal inflammatory response was present in 85.8% and fetal inflammatory response in 73.8%, with funisitis in 33.8% (Table 4). We next examined outcomes among pregnancies with pathogenic cultures, stratified by the presence or absence of histologic inflammation. Co-occurrence of histopathologic evidence of inflammation with isolation of a pathogenic isolate from the placenta had high rates of maternal and neonatal morbidity and neonatal sepsis (Figure 3). Several pathogens were associated with high rates (>70%) of histopathologic inflammation, including GBS, *Haemophilus* species, *Klebsiella* spp, *Prevotella* spp, *Staphylococcus aureus*, and *Fusobacterium* (Figure 3*C*). Within the nonpathogenic group, only specimens labeled “normal vaginal flora” were associated with histologic evidence of chorioamnionitis (Figure 3*D*). The absence of histologic inflammation was associated with significantly earlier gestational age at delivery (172 vs 207 days; *P* < .001) and a markedly higher rate of stillbirth (53.9% vs 9.3%, *P* < .001) (Table 4 and Figure 3*E*), suggesting that alternative, noninfectious causes contribute to these outcomes in the absence of inflammation of the placenta.

**Table 4.** Pathogenic Placental Cultures With Evidence of Histopathologic Chorioamnionitis

Outcome	Histopathologic Chorioamnionitis, % (No.) (n = 335)	No Histopathologic Chorioamnionitis, % (No.) (n = 128)	*P* Value
Demise at >14 wk GA	9.3 (31)	53.9 (69)	<.001^a^
Suspected IUI^b^	49.3 (164)	11.7 (15)	<.001^a^
Antenatal antibiotics	69.2 (231)	35.2 (45)	<.001^a^
Maternal hemorrhage^c^	19.4 (65)	14.1 (18)	.18
Endometritis or wound infection^d^	11.9 (40)	2.5 (3)	.002^a^
Maternal ICU admission	6.34 (18)	3.96 (4)	.38
Maternal death	0.35 (1)	0	.55
Composite adverse maternal outcome^e^	31.9 (107)	17.2 (22)	.002^a^
Neonatal demise	9.1 (19)	5.6 (2)	.49
Neonatal antibiotics	99.5 (190)	73.5 (25)	<.001^a^
Neonatal sepsis^f^	61.2 (134)	28.2 (11)	<.001^a^
Culture-positive neonatal sepsis^g^	28.7 (50)	8.8 (3)	.01^a^
Composite adverse neonatal outcome^h^	84.3 (183)	52.8 (19)	<.001^a^

Abbreviations: GA, gestation age; ICU, intensive care unit; IUI, intrauterine infection.

^a^Significant at *P* < .05.

^b^Suspected IUI as documented in clinically by nursing or physician staff.

^c^Maternal hemorrhage was defined as administration of >1 uterotonic and/or estimated blood loss >1000 mL.

^d^Pregnancy complicated by a clinical diagnosis of postpartum endometritis or wound complications defined as wound dehiscence, cellulitis, or wound infection requiring treatment.

^e^Composite adverse maternal outcome defined as postpartum wound infection with hospital admission, intra-abdominal abscess, endometritis with hospital admission, postpartum reoperation, need for >1 uterotonic agent, intrauterine balloon placement, ICU admission, mechanical ventilation or vasopressor support, and/or maternal death.

^f^Neonatal sepsis occurring at <7 days of life, defined as a positive blood culture or treatment of neonatal sepsis with >7 days of antibiotics initiated within 48 hours of birth.

^g^Neonatal sepsis occurring at <7 days of life with a positive neonatal blood culture.

^h^Composite adverse neonatal outcome defined as unexpected neonatal ICU admission (eg, in a term neonate), neonatal sepsis defined as positive blood cultures or treatment with >7 days of antibiotics initiated within 48 hours of birth, necrotizing enterocolitis, or neonatal death.

### Emerging or Underrecognized Pathogens and Disproportionate Adverse Outcomes

**Figure 4.**
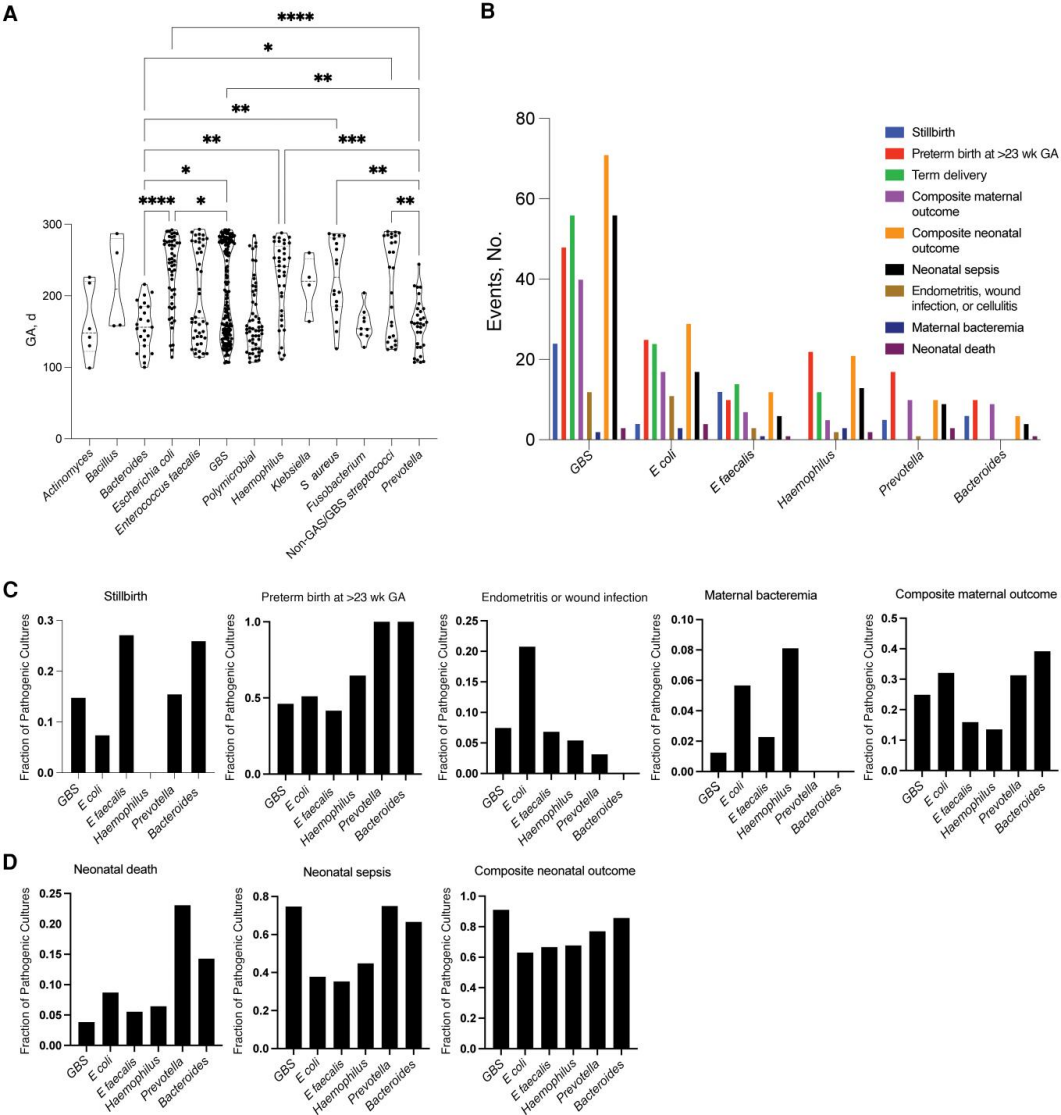
Comparison of pregnancy outcomes by bacterial pathogen. *A,* Gestational age (GA) at delivery by pathogenic culture result (n = 477). *P < .05, **P < .005, ***P < .0005, ****P < .0001. *B,* Frequency of adverse outcomes by pathogen result. *C,* Frequency of adverse maternal outcomes associated with the most frequently isolated bacterial pathogens, including stillbirth (n = 51), preterm birth (n = 132), endometritis (n = 29), maternal bacteremia (n = 9), and composite adverse maternal outcome (n = 88). *D,* Frequency of neonatal outcomes with the most frequently isolated bacterial pathogens, including neonatal death (n = 14), neonatal sepsis (n = 105), and composite adverse neonatal outcome (n = 149). Abbreviations: GAS, group A *Streptococcus*; GBS, group B *Streptococcus*.

As a secondary objective, we explored whether specific bacterial pathogens isolated from placental cultures were associated with disproportionately adverse maternal or neonatal outcomes. To investigate this, we stratified maternal and neonatal outcomes by the most common pathogenic organisms, including GBS, *E coli*, *E faecalis*, *Haemophilus* spp, *Bacteroides* spp, and *Prevotella* spp (Figure 4). Gestational age at delivery varied significantly by pathogen (*P* < .001) (Figure 4*A*). Specifically, pregnancies with placental cultures with *Bacteroides* or *Prevotella* spp were delivered at lower gestational ages than those with GBS, *E coli*, *Haemophilus*, and other streptococcal species. Neonatal outcomes were also different: *Prevotella* was associated with the highest rate of neonatal death, and both *Bacteroides* and *Prevotella* were linked to high rates of neonatal sepsis (>60%). GBS cultures were associated with the highest rates of composite neonatal morbidity, reflecting both its frequency and severity. For maternal outcomes, *E coli* was associated with high rates of endometritis and wound infections, and *Haemophilus* spp was associated with high rates of maternal bacteremia.

### Polymicrobial Cultures

**Figure 5.**
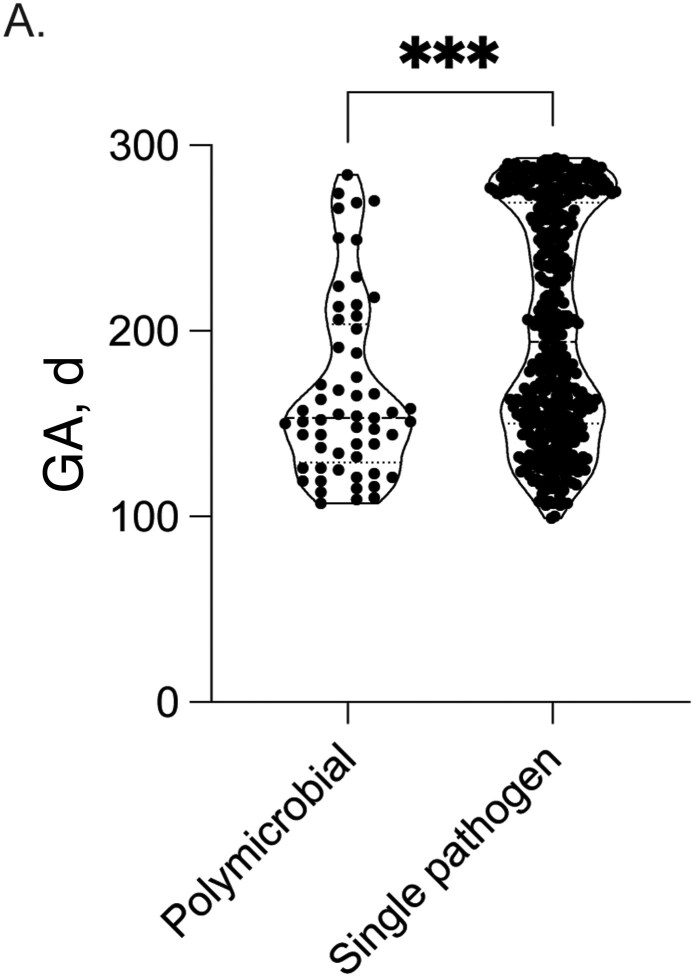
Gestational age (GA) at delivery by polymicrobial pathogenic culture compared with single bacterial pathogen in placental culture. ***P < .001.

Previous studies have demonstrated that ascending infection is often polymicrobial [10, 28]. The overall frequency of polymicrobial cultures (11.5% of pathogenic cultures) was lower than what was previously reported as associated with ascending infection and chorioamnionitis [10, 32]. The composition of polymicrobial pathogenic cultures was diverse and is represented in Supplementary Tables 5 and 6. Polymicrobial results most commonly involved combinations of *E faecalis/E coli* and GBS*/S aureus*. *Haemophilus* in polymicrobial cultures was exclusively identified with GBS. The gestational age at delivery was significantly lower with polymicrobial than with single pathogens (167.7 vs 202.3 days, respectively; *P* < .001) (Figure 5). There were no significant differences in composite maternal or neonatal outcomes associated with polymicrobial cultures.

### Antimicrobial Resistance Patterns of Placental Pathogens

Current American College of Obstetricians and Gynecologists guidelines suggest that empiric treatment of suspected intrauterine should be designed by “consulting local microbiology laboratory and infectious disease experts to ascertain whether there are alternative recommended regimens based on local antibiotic resistance patterns” [33]. Therefore, we assessed pathogen susceptibility to empiric regimens (Table 5). More than 30% of *E faecalis*, *Bacteroides*, and *Haemophilus* isolates would not be adequately covered by ampicillin with gentamicin. While *S aureus* was a less frequent isolate (in 5.1% of all cultures), 29% of those isolates were methicillin-resistant *S aureus*, and 62.5% were clindamycin resistant. Of *E coli* isolates, 42.2% were resistant to ampicillin-sulbactam and 4.4% to gentamicin. The gentamicin-resistant *E coli* also had universal resistance to ampicillin and ampicillin-sulbactam but was susceptible to both cefoxitin and cefotetan. Notably, 7.4% of *E coli* isolates were ESBL producing. Placental cultures yielding ESBL-producing *E coli* were associated with neonatal infection by ESBL *E coli* strains, indicating likely vertical transmission. These findings underscore the growing relevance of resistant gram-negative organisms in the obstetric population.

**Table 5.** Antimicrobial Resistance in the Cohort

Regimen	Pathogenic Cultures Covered, % (No.)	% Unknown^a^
Ampicillin-gentamicin	85.4 (388)	7.7
Cefazolin-gentamicin	61.7 (291)	3.6
Clindamycin-gentamicin	47.1 (137)	40.8
Cefotetan	81.2 (362)	9.3
Cefoxitin	82.5 (368)	9.3
Vancomycin-gentamicin	75.6 (362)	2.6
Ampicillin-sulbactam	87.8 (355)	5.3

^a^Sensitivities not routinely performed or not performed on the bacterial isolate. Includes group B *Streptococcus* for clindamycin, *Bacteroides* for cefoxitin or cefotetan if annotated β-lactamase positive, and *Bacillus* for β-lactam antibiotics.

## DISCUSSION

In this large retrospective cohort of high-risk pregnancies, we characterized the microbial landscape of placental cultures and associated clinical outcomes in the modern antimicrobial era. This cohort had a high rate of adverse maternal and neonatal outcomes, reflecting clinical outcomes prompting placental culture at our institution. ICU admission rates for both the pathogenic and nonpathogenic groups are higher than the Centers for Disease Control and Prevention’s nationally reported maternal rate during 2020–2022 [34]. The rate of maternal sepsis (3.6% overall) is also higher than in other studies [35, 36], reflecting the high morbidity rate in this cohort.

Our findings demonstrate that GBS and *E coli* remain predominant obstetric pathogens. Universal GBS screening and intrapartum prophylaxis is routinely implemented in this hospital system, and antibiotics were administered prior to delivery (and collection of placental cultures) in 61% of cases with pathogenic and 56% of cases of nonpathogenic isolates. Of note, 82% of patients with GBS in a placental culture were treated with a β-lactam before delivery. Despite this, GBS remained the most common isolate and was associated with severe maternal morbidity, including the only maternal death and 2 of 3 puerperal hysterectomies (both secondary to infection). The continued prevalence of GBS, along with high concordance between placental and neonatal blood cultures, demonstrates the persistent vertical transmission risk and highlights the need for additional prevention strategies beyond intrapartum prophylaxis, such as maternal vaccination.

### Placental Culture as a Surveillance Tool

Current diagnostic approaches to intrauterine infection are limited. Placental culture may provide clinically meaningful information. In our cohort, pathogens isolated from placental tissue were correlated with both histopathologic chorioamnionitis and clinical morbidity. This supports the utility of placental culture as a surveillance tool for emerging pathogens and antimicrobial resistance trends at the maternal-fetal interface.

### Emerging and Underrecognized Pathogens

This study also identified several underrecognized or emerging pathogens with meaningful clinical impact. *E faecalis* and *Haemophilus* spp were frequently isolated and associated with adverse outcomes, despite limited representation in the literature [33–36]. *Prevotella* and *Fusobacterium* spp, previously considered components of normal vaginal flora with limited association with adverse outcomes [37–40], were reclassified as pathogenic based on associations with early delivery and histologic inflammation. These organisms were associated with distinct clinical patterns: *Prevotella* and *Bacteroides* were linked to the earliest gestational ages at delivery and high rates of neonatal sepsis, while *Haemophilus* spp exhibited a predilection for maternal bacteremia. These findings suggest that the microbial landscape of ascending infection may be broader and more dynamic than historically appreciated.

### Antimicrobial Resistance and Implications for Empiric Therapy

A central objective of this study was to evaluate the antimicrobial susceptibility of placental pathogens in the context of current empiric therapy recommendations. Surveillance of antimicrobial resistance is recommended for developing empiric regimens for the mother-infant dyad [14, 15, 41–43]. We observed substantial antimicrobial resistance across multiple organisms: including 42% ampicillin-sulbactam resistance and 7.4% ESBL production among *E coli.* We also observed >50% clindamycin resistance among tested GBS (testing is not uniformly performed unless requested) and 29% of clindamycin resistance in *S. aureus isolates*. These data are concordant with national studies demonstrating rising resistance in these isolates, including in maternal and neonatal populations [20, 42, 44–46].

These resistance profiles raise concerns about the adequacy of some empiric regimens such as ampicillin-gentamicin or clindamycin-based therapies in penicillin-allergic patients. In contrast, recommended second-generation cephalosporin regimens had >80% of coverage in this cohort. Given that >10% of pathogenic cultures were polymicrobial and many organisms exhibited multidrug resistance, broader-spectrum empiric coverage may warrant consideration in select high-risk populations. These data further support a thorough history to accurately characterize antibiotic allergies, along with the use of penicillin allergy testing and graded challenges to allow for appropriate coverage of these pathogens. In addition, these findings reinforce the importance of antimicrobial resistance surveillance specific to the obstetric population to inform evidence-based updates for treatment guidelines. However, given the single-center study design, any changes to empiric therapy should require validation in larger, multicenter cohorts.

### Strengths and Limitations

This study has several strengths, including its large sample size, linkage of microbiologic, clinical, and histopathologic data, and inclusion of antimicrobial susceptibility testing. The use of placental pathology allowed correlation of microbiologic findings with objective inflammatory responses, strengthening the biological plausibility of associations observed. The primary limitation is the retrospective design, which precludes causal inference and is influenced by the high prevalence of antepartum antibiotic exposure. A key limitation of this study is that placental cultures were obtained only in high-risk clinical scenarios and were available for analysis only with a positive microbial result, leading to the absence of a “denominator” population—that is, the population in which placental cultures were collected in a high-risk clinical scenario but had no growth. As such, we cannot determine the overall prevalence of culture positivity or compare outcomes with those in culture-negative cases. Although including nonpathogenic placental isolates (eg, *Lactobacillus*) as a comparison facilitated a reduction in bias, selection bias remains, and our findings should be interpreted as associations within a high-risk cohort rather than population-level estimates. Nonetheless, the consistency, magnitude, and directionality of associations across multiple outcomes support the clinical relevance of the pathogens identified.

Studies using rigorous contamination-controlled sequencing approaches have shown that placental tissue from healthy pregnancies contains minimal bacterial signal. Notably, Kennedy et al [23] and others [24, 25, 47–49] demonstrated that bacterial signal in placental tissue obtained from healthy pregnancies is minimal and often attributable to contamination during sampling. These findings suggest that organisms identified in placental cultures reflect microbial exposure. It is important to note that our study does not aim to define a placental microbiome but rather to characterize clinically relevant organisms present at the maternal-fetal interface in high-risk pregnancies with increased probability of infection. Because organisms were identified with traditional bacterial cultures rather than molecular methods, some exposure during sampling is possible. However, the strong association between specific bacteria, histopathologic inflammation, and adverse outcomes suggests that these organisms contribute meaningfully to maternal and neonatal morbidity.

Although placental cultures are obtained in a context where contamination is possible, given the proximity to the colonized lower gastrointestinal and genitourinary tracts, several observations suggest that these findings reflect clinically relevant microbial exposure rather than random contamination alone. First, the strong association with histopathologic inflammation supports a biologically meaningful host response rather than incidental colonization. Second, specific organisms were consistently associated with adverse maternal and neonatal outcomes, supporting an association with clinically relevant pathology. Third, placental cultures yielding microbial growth represent a relatively small and clinically selected subset of deliveries in our institution (which performs approximately 10 000 deliveries per year), supporting a nonrandom process of microbial detection. Together, these findings support the interpretation that placental cultures in this context capture clinically meaningful microbial exposure at the maternal-fetal interface, even if the precise origin of organisms cannot be definitively determined. These data highlight the need for additional studies understanding the complex host-pathogen interactions that lead to ascending infection and contributing to morbidity in this population.

### Conclusions

In summary, this study provides an updated characterization of pathogens at the maternal-fetal interface and identifies significant antimicrobial resistance among organisms associated with intrauterine infection. These findings underscore the value of placental cultures as a surveillance tool and highlight the need for modernized empiric antibiotic regimens and ongoing antimicrobial resistance monitoring in obstetric populations. Strengthening microbiologic surveillance and refining clinical algorithms may improve maternal and neonatal outcomes in settings where infection remains a major driver of morbidity.

## Supplementary Material

ofag229_Supplementary_Data

## Supplementary Data

Supplementary materials are available at *Open Forum Infectious Diseases* online. Consisting of data provided by the authors to benefit the reader, the posted materials are not copyedited and are the sole responsibility of the authors, so questions or comments should be addressed to the corresponding author.
