# Supplementary material for: Placental Pathogens Associated With Adverse Maternal and Neonatal Outcomes
Source: Open Forum Infect Dis. 2026 Apr 22;13(6):ofag229. doi: 10.1093/ofid/ofag229 (PMC13270239; doi:10.1093/ofid/ofag229)
Supplement: ofag229_Supplementary_Data [file ofag229_supplementary_data.docx]

**Supplemental Figure 1**

**
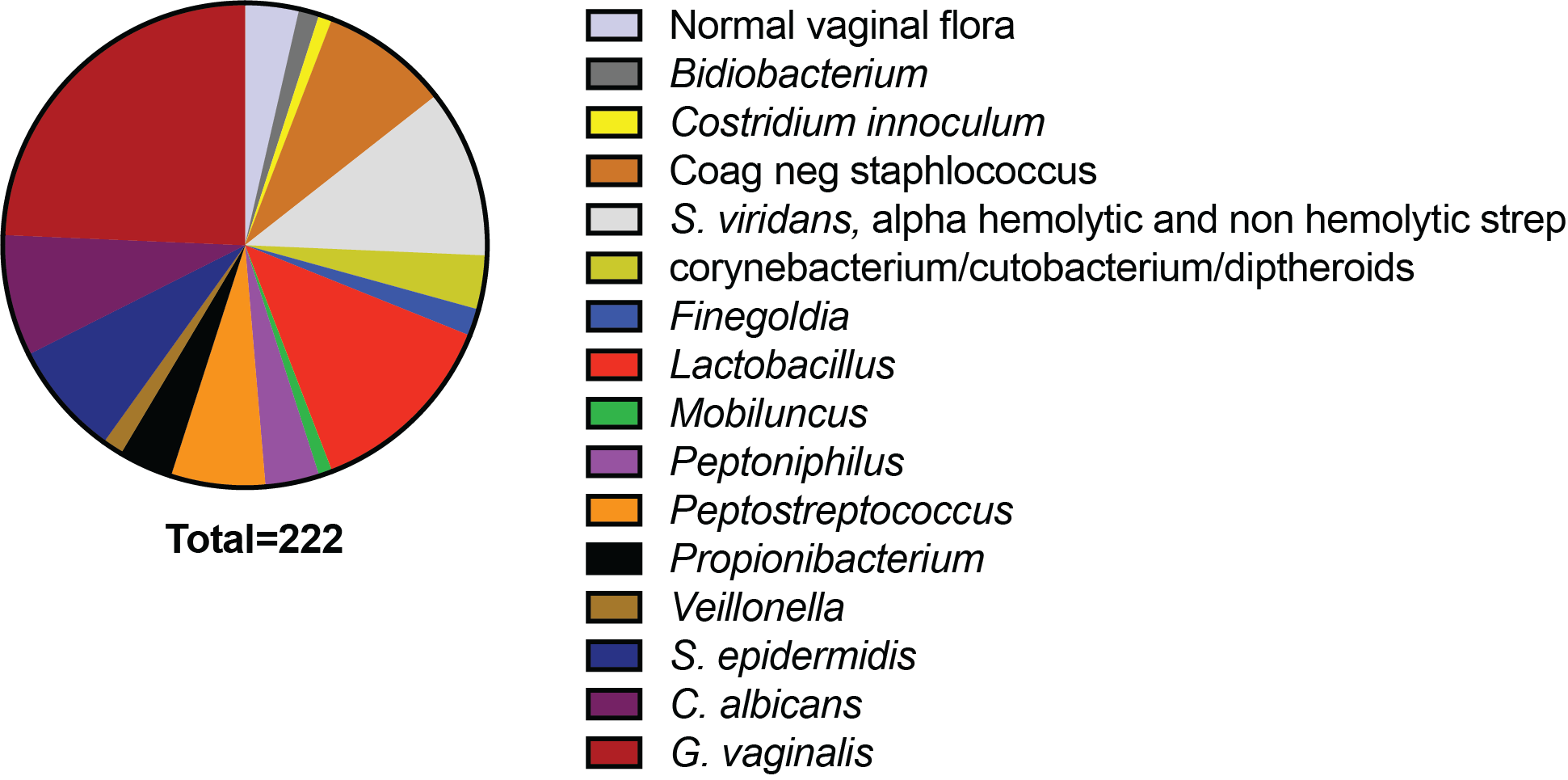
**

Supplemental Figure 1. Frequency of bacteria categorized as non-pathogenic in placental cultures

**Supplemental Table 1 Data Abstracted from the Clinical Chart**

Maternal Data

| Placental Pathology |
| --- |
| Penicillin Allergy |
| Age |
| Gravida/Parity |
| Prior Preterm Birth |
| Indication For Cx |
| IUFD/Stillbirth |
| Clinical Diagnosis Of Suspected Chorioamnionitis |
| PPROM |
| Length Of PPROM |
| Length Of Time From Admission To Delivery |
| Gestational Age At PPROM |
| Gestational Age At Delivery Or Completion Of Pregnancy |
| Antibiotics Prior To Delivery |
| Indication For Antibiotic: Surgical prophylaxis, GBS prophylaxis, Suspected chorioamnionitis, sepsis |
| All Antibiotics Given (List) |
| Sepsis |
| Postpartum Antibiotics |
| Indication For Postpartum Antibiotics |
| Any Maternal Blood Cultures Sent (Antepartum Or Postpartum) |
| Blood Culture Result |
| Mode Of Delivery |
| Induction Of Labor |
| Indication For Induction |
| Indication For Cesarean Delivery |
| Postpartum Wound Complications (Endometritis, Cellulitis) |
| Postpartum Reoperation |
| Indication For Reoperation (Retained Placenta, POC) |
| Other Complications: Hysterectomy, Hemorrhage > 1000ml |
| >1 Uterotonic Administration Post Delivery (Everyone Gets Pit. Misoprostol, Methergine, Hemabate, Txa) |
| Balloon Placement (JADA/Bakri) For Hemorrhage |
| ICU Admission |
| Intubation/Pressor Support |
| Maternal Death |

Neonatal Data

| >23 Weeks Born Alive |
| --- |
| Neonatal Demise |
| Supportive Measures Only |
| NICU Admission |
| Neonatal Complications |
| NEC |
| Neonatal Sepsis |
| Positive Neonatal Cultures |
| Neonatal Culture Antimicrobial Resistance |
| Neonatal Antibiotics |
| Length Of Neonatal Antibiotics |

**Supplemental Table 2 Organisms identified on placental culture**

| Organism | n | Percent total |
| --- | --- | --- |
| GBS | 161 | 22.5 |
| polymicrobial | 57 | 8.0 |
| *Gardnerella vaginalis* | 54 | 7.6 |
| *E. coli* | 53 | 7.4 |
| *E. faecalis* | 44 | 6.2 |
| *Haemophilus sp.* | 37 | 5.2 |
| *Prevotella* | 32 | 4.5 |
| *Lactobacillus sp.* | 29 | 4.1 |
| *S. anginosus, mitis, gallolyticus* | 26 | 3.6 |
| *Non pathogenic streptococcus* | 25 | 3.5 |
| *Bacteroides* | 23 | 3.2 |
| Coagulase negative *Staphlococci* | 19 | 2.7 |
| *S. aureus* | 18 | 2.5 |
| *S. epidermidis* | 17 | 2.4 |
| *C. albicans* | 17 | 2.4 |
| *Peptostreptococcus* | 14 | 2.0 |
| *Fusobacterium* | 9 | 1.3 |
| *Peptoniphilus* | 8 | 1.1 |
| normal vaginal flora | 8 | 1.1 |
| *Propionibacterium* | 8 | 1.1 |
| *Actinomyces* | 6 | 0.8 |
| Diptheroids/*Corynebacterium* | 5 |  |
| *Finegoldia* | 4 |  |
| *Bacillus cereus* | 4 |  |
| *Klebsiella sp.* | 4 |  |
| *Pseudomonas aeruginosa* | 3 |  |
| *Bifidobacterium* | 3 |  |
| *Cutibacterium* | 3 |  |
| *Veilonella* | 3 |  |
| *Mycoplasma hominis* | 2 |  |
| *Clostridium innoculum* | 2 |  |
| *Mobiluncus* | 2 |  |
| *Citrobacter* | 2 |  |
| *Listeria* | 2 |  |
| *Proteus mirabilis* | 2 |  |
| *Capnocytophaga* | 1 |  |
| *Eikenella* | 1 |  |
| Group A Streptococcus | 1 |  |
| Pneumococcus | 1 |  |
| *Serratia marcescens* | 1 |  |
| *Enterobacter cloacae* | 1 |  |
| *Achromobacter* | 1 |  |
| *C. glabrata* | 1 |  |

**Supplemental Table 3 Neonatal Culture Results with Concordant Cultures and polymicrobial cultures**

| **Placental Culture** | **Neonatal Culture** |
| --- | --- |
| *S. aureus* | *K. pneumoniae, S. aureus ** |
| *E. coli* | GBS, *E. coli** |
| *E. faecalis* | *Kuyvera* sp., GBS *E. faecalis** |
| *S. anginosus* | *P. aeruginosa, K. pneumoniae* |
| *S. aureus,* GBS, *Citrobacter koseri* * | *Citrobacter koseri* |
| *Bacteroides ovatus, Citrobacter koseri*, E. faecalis* | *Citrobacter koseri* |

*included in concordant cultures

**Supplemental Table 4 Neonatal Culture Results with Discordant Placental Cultures**

| **Placental Culture** | **Neonatal Culture** |
| --- | --- |
| *S. aureus* | *S. epidermidis* |
| *S. aureus* | Coagulase negative staphylococcus |
| *S. mitis, S. anginosus* | *K. pneumoniae, Pseudomonas aeruginosa* |
| Prevotella | *S. epidermidis* |
| Prevotella | Coagulase negative staphylococcus |
| S. gallolyticus | E. faecalis |
| *B. fragilis* | *S. hominis* |
| *Actinomyces neuii* | *E. coli* |
| *M. hominis* | Coagulase negative staphylococcus |
| *E. coli* | Coagulase negative staphylococcus |
| *Diptheroids* | *Brevibacterium* |
| *Lactobacillus jensenii* | *Klebsiella pneumoniae* |
| *Lactobacillus jensenii* | *corynebacterium* |
| *Peptostreptococcus* | *Coagulase negative Staphylococcus* |
| *Peptostreptococcus* | *MRSA* |
| *Alpha hemolytic streptococci* | *S. epidermidis* |
| *S. viridans* | *S. anginosus* |
| *S. viridans* | *S. epidermidis* |
| *S. epidermidis* | *E. coli* |
| *C. albicans* | *S. epidermidis* |
| *G. vaginalis* | *S. epidermidis* |
| *G. vaginalis* | *S. hominis* |
| *G. vaginalis* | *S. anginosus* |
| *G. vaginalis* | *Coagulase negative Staphylococcus* |

**Supplemental Table 5 Polymicrobial culture Combinations**

| **Bacteria species** | **Co-culture species 1** | **Co-culture species 2** | **Co-culture species 3** |
| --- | --- | --- | --- |
| *B. ovatus* | *C. koseri* | *E. faecalis* | *Actinomyces* |
| GBS | *B. Vulgatus* | *B. Uniformis* |  |
| GBS | *E. faecalis* | *S. aureus* |  |
| GBS | *S. aureus* | *E. coli* |  |
| GBS | *H. parainfluenzae* | *S. aureus* |  |
| GBS | *E. faecalis* | Klebsiella |  |
| GBS | *Citrobacter koseri* | *S. aureus* |  |
| GBS | *B. vulgaris* | *B. uniformis* |  |
| *B. ovatus* | *Citrobacter koseri* | *E. faecalis* |  |
| *E.coli* | *B. uniformis* | *B. caccae* |  |
| GBS | *E.coli* |  |  |
| GBS | *C. perfringens* |  |  |
| GBS | *E. faecalis* |  |  |
| GBS | Bacteroides |  |  |
| GBS | *H. parainfluenzae* |  |  |
| GBS | *N. gonorrhoeae* |  |  |
| GBS | *P. aeruginosa* |  |  |
| GBS | *S. aureus* |  |  |
| GBS | Prevotella |  |  |
| *E.coli* | Bacteroides sp. |  |  |
| *E.coli* | *E. feacalis* |  |  |
| *E.coli* | *M. morganii* |  |  |
| *E.coli* | *P. mirabilis* |  |  |
| *S. pyogenes* | *E.coli* |  |  |
| *Actinomyces* | Prevotella |  |  |
| *K. pneumoniae* | *E. aerogenes* |  |  |
| Prevotella | *S. anginosus* |  |  |
| Prevotella | *S. gallolyticus* |  |  |
| *E. faecalis* | *Klebsiella* |  |  |
| *E. faecalis* | *P. mirabilis* |  |  |
| *Mycoplasma hominis* | *B*acteroides |  |  |
| *S. aureus* | *E. feacalis* |  |  |

**Supplemental Table 6 Co-occurrence of bacterial species in placental polymicrobial culture**

| isolate A | isolate B | n | % total | % isolate A | % isolate B |
| --- | --- | --- | --- | --- | --- |
| GBS | Bacteroides | 3 | 5.3 | 10.7 | 21.4 |
| GBS | *E. coli* | 5 | 8.7 | 17.9 | 21.7 |
| GBS | *S. aureus* | 9 | 15.7 | 32.1 | **90.0** |
| GBS | Haemophilus | 5 | 8.7 | 17.9 | **100.00** |
| GBS | *E. faecalis* | 5 | 8.7 | 17.9 | 29.4 |
| GBS | other* | 7 | 8.89 | 25.0 |  |
| *E. coli* | Bacteroides | 6 | 10.5 | 26.0 | 42.8 |
| *E. coli* | *E. faecalis* | 9 | 15.7 | 39.1 | **52.9** |
| *E. coli* | other** | 4 | 7.0 | 17.3 |  |
| *E. faecalis* | Klebsiella sp. | 3 | 5.3 | 17.6 | **60.0** |
| *E. faecalis* | *S. aureus* | 3 | 5.3 | 17.6 | 30.0 |

**C. perfringens, P. aeruginosa, N. gonorrhoeae, K. pneumoniae, Prevotella, Citrobacter sp*. ***S. pyogenes, M. morganii, P. mirabilis, S. aureus*
